# Role of MUC20 overexpression as a predictor of recurrence and poor outcome in colorectal cancer

**DOI:** 10.1186/1479-5876-11-151

**Published:** 2013-06-20

**Authors:** Xiuying Xiao, Lisha Wang, Ping Wei, Yayun Chi, Dali Li, Qifeng Wang, Shujuan Ni, Cong Tan, Weiqi Sheng, Menghong Sun, Xiaoyan Zhou, Xiang Du

**Affiliations:** 1 Department of Pathology, Fudan University Shanghai Cancer Center, Shanghai, 200032, China; 2 Department of Oncology, Shanghai Medical College, Fudan University, Shanghai, 200032, China; 3 Institute of Pathology, Fudan University, Shanghai, 200032, China; 4 Institutes of Biomedical Sciences, Fudan University, Shanghai, 200032, China; 5 Department of Oncology, Renji Hospital, School of Medicine, Shanghai Jiaotong University, Shanghai, 200127, China; 6 Breast Cancer Institute, Fudan University Shanghai Cancer Center, Shanghai, 200032, China

**Keywords:** MUC20, Colorectal Cancer, Invasion, Recurrence

## Abstract

**Background:**

Colorectal cancer (CRC) remains one of the most common cancers worldwide. We observed that MUC20 was significantly up-regulated in CRC patients with poor prognosis based on the microarray analysis. However, little is known about the role of MUC20 in CRC.

**Methods:**

Microarray experiments were performed on the Affymetrix U133 plus 2.0 GeneChip Array. The protein and mRNA levels of MUC20 were examined by immunohistochemistry (IHC) and Real-Time quantitative PCR (RT-qPCR) in CRC tissues and adjacent noncancerous tissues (ANCT). ShRNA and overexpression plasmids were used to regulate MUC20 expression in CRC cell lines in vitro; wound healing, Transwell migration assays, and Western blotting were used to detect migration and invasion changes.

**Results:**

MUC20 was one of the up-regulated genes in CRC patients with poor prognosis by microarray. Using IHC and RT-qPCR, we showed that MUC20 expression was significantly higher in CRC tissues than in ANCT (P < 0.05). We further showed that MUC20 overexpression was correlated with recurrence and poor outcome (P < 0.05). The Kaplan-Meier survival curves indicated that disease-free survival (DFS) and overall survival (OS) were significantly worse in CRC patients with MUC20 overexpression. The Cox multivariate analysis revealed that MUC20 overexpression and TNM stage were independent prognostic factors. Elevated expression of MUC20 in cells promoted migration and invasion, whereas ShRNA-mediated knockdown inhibited these processes. In addition, Western blotting demonstrated that MUC20-induced invasion was associated with MMP-2, MMP-3, and E-cadherin.

**Conclusions:**

Cumulatively, MUC20 may serve as an important predictor of recurrence and poor outcome for CRC patients. MUC20 overexpression could enhance migration and invasion abilities of CRC cells. Translation of its roles into clinical practice will need further investigation and additional test validation.

## Background

CRC is one of the most common cancers, with poor prognosis, and accounts for almost half a million deaths annually worldwide. Death of these patients results from uncontrolled metastatic disease, including peritoneum metastases, lymph nodes metastases, liver metastases, and so on [1-3]. Tumor metastasis is responsible for approximately 90% of all cancer-related deaths [4,5]. The molecular pathogenesis and progression of CRC is complicated and poorly understood.

Mucins are large extracellular glycoproteins that are heavily glycosylated with complex oligosaccharides and are produced by epithelial cells [6,7]. The core proteins for human mucins (MUC1-MUC8, MUC12, MUC13, MUC15-17, and MUC19-21) have been identified [8]. Many mucins, including MUC1, MUC2, MUC4, and MUC5AC, were abnormally expressed and aberrantly glycosylated in adenocarcinomas, and were associated with carcinogenesis, tumor invasion, and a poor patient outcome [9-11]. Previous studies found that up-regulation of MUC1, down-regulation of MUC2, and up-regulation of MUC5AC are all involved in the development and progression of CRC [12-14].

Based on the whole-genome expression profiling of CRC, we observed that MUC20, a newly recognized biomarker, was significantly up-regulated in CRC patients with poor prognosis. MUC20 was first recognized as an up-regulated novel mucin protein in Immunoglobulin A nephropathy (IgAN) patients [15]. In IgAN, MUC20 was a negative regulator of the Met signaling cascade, which had a role in suppression of the Hepatocyte Growth Factor-Induced Grb2-Ras pathway [16].

Although many mucins play crucial roles in tumor development, no association of MUC20 with CRC has been reported. In this study, we evaluated MUC20 mRNA/protein expression to determine its prognostic significance in CRC patients. Furthermore, we analyzed functions of MUC20 in CRC cell lines by transfection in vitro.

## Materials and methods

### Patient samples

81 RNAlater-preserved CRC tissues and another 51 paired RNAlater-preserved CRC tissues and ANCT were obtained from Fudan University Shanghai Cancer Center. Additionally, 150 formalin-fixed paraffin-embedded (FFPE) CRC tissues and ANCT were obtained from the pathology archives of our Cancer Center. Cancers were assessed according to the WHO classification. The inclusion criteria were as follows: primary sporadic colorectal adenocarcinoma (excluding mucinous carcinoma); aged from 18 to 75 years; no preoperative chemotherapy and radiotherapy; similar postoperative chemotherapy regimens. This study was approved by the Ethical Committee of our Cancer Center, and written informed consent was obtained from each patient.

### Cell culture and reagents

The human CRC cell lines HCT-116, LoVo, and SW620 were cultured in DMEM (GIBCO BRL) supplemented with 10% FBS, 100 units/mL penicillin, and 100 μg/mL streptomycin. The human CRC cell line SW480 was cultured in RPMI 1640 (GIBCO BRL) supplemented with 10% FBS, 100 units/mL penicillin, and 100 μg/mL streptomycin. All cells were cultured in a 5% CO_2_ incubator at 37°C. The primary antibody MUC20 was purchased from Abcam; MMP3 and E-cadherin were purchased from Epitomics; MMP2 was purchased from Bioworld. The secondary antibodies (horseradish peroxidase–linked anti-mouse immunoglobulin G, and anti-rabbit immunoglobulin G) were purchased from Cell Signaling Technology.

### Microarray analysis

Total RNA was extracted from 81 CRC tissues using TRIzol reagent (Invitrogen). RNA concentration was assessed with a NanoVue spectrophotometer and RNA integrity was verified using an Agilent 2100 bioanalyzer. Gene expression profiling of 81 CRC tissues was performed by Affymetrix GeneChip Human Genome U133 Plus 2.0 array platform. Data reading was performed using QuantArray R software, and data analysis was performed using Cluster3.0 and SAM2.0 software. After normalization against the control gene (GAPDH), a gene was designated as differential if expression in patients with poor prognosis was > =1.5-fold than in patients with good prognosis.

### Tissue microarray and IHC

Tissue microarray (TMA) was constructed using the specimens from 150 paraffin-embedded blocks of CRC primary tumors and ANCT. Two CRC tissue cores and two ANCT cores from the same case were arranged on a recipient paraffin block (1 mm core). IHC was performed using the Envision System with diaminobenzidine (Gene Tech, Shanghai Company Limited) according to the manufacturer’s protocol. The working concentration of MUC20 mouse anti-human polyclonal antibody was 1:100. A granular cytoplasmic stain was considered as positive. PBS was used as a negative control.

Tissue microarray slides were blindly evaluated by two of the authors twice. A staining index (SI, range 0-9) was used to evaluate the results with the following formula: SI = intensity * positive area, where intensities were scored as 0 (negative), 1+ (faint/equivocal), 2+ (moderate), and 3+ (strong). Immuno-positive areas were categorized as 0 (0%), 1 (<10%), 2 (10-50%), 3 (>50%). When SI ranged from 0 to 2, the results were defined as negative. If the SI ranged from 3 to 9, the results were defined as positive [17]. If there are two cores did not yield identical immunostaining, compare them with the whole-section and select the consistent one.

### RNA extraction and RT-qPCR

Total RNA was extracted from 51 pairs of CRC tissues and ANCT, and cultured CRC cells using TRIzol reagent (Invitrogen). Quantitative analysis of MUC20 mRNA expression was performed in CRC tissues, ANCT, and in four CRC cell lines. MUC20 was amplified with the following primes: 5’-CAA GAT CAC AAC CTC AGC GA -3’ (forward primer) and 5’-ACC TCC ATT TTC ACC TGC AC-3’ (reverse primer). GAPDH was used as an endogenous control with the following primers: 5’-GAA AGT CCG GAA GTC TCT GG-3’ (forward primer) and 5’-TAG AGA CTT GGG CAG TGT GG-3’ (reverse primer). The cycling conditions for MUC20 and GAPDH were as follows: one cycle of 95°C for 5 minutes; 40 cycles of 95°C for 20 seconds, 58°C for 30 seconds, and 68°C for 45 seconds; and one cycle of 72°C for 10 minutes. The specificity of the PCR amplification was validated by a single peak in the melting curves. Each RT-qPCR experiment was repeated three times.

### Plasmid construction and transfection

The pGPU6/GFP/Neo expression vector was purchased from Shanghai GenePharma Co. Ltd. The interfering oligonucleotide designed with a short hairpin structure targeting MUC20 was cloned into the pGPU6/GFP/Neo vector. The recombinant vector pGPU6/GFP/Neo-shRNA-MUC20 was confirmed by DNA sequencing and enzyme digestion analysis.

The pIRES2-EGFP expression vector was purchased from Shanghai R&S Biotechnology. The full-length encoding cDNA of MUC20 (GenBank accession number NM_001098516.1) was generated by PCR. The PCR product and the pIRES2-EGFP plasmid vector were double digested with EcoRI/SalI enzymes and then ligated to each other using T4 DNA ligase. The recombinant vector pIRES2-EGFP-MUC20 was confirmed by DNA sequencing and enzyme digestion analysis. The recombinant vectors were transfected into CRC cell lines using FuGENE® HD Transfection Reagent (Roche) according to the manufacturer’s instructions.

### Wound healing assay

LoVo and SW620 cells were seeded into 6-well plates, cultured until 80% confluent, artificial wounds were gently made in the plate using a micropipette tip, and the cells were washed with serum-free medium to remove floating cells and debris. Representative images of cells migrating into the wounds were captured at 0 hour and 48 hours in the same wounded region using an inverted microscope.

### Transwell assay

For the invasion assays, Transwell inserts with an 8-μm pore size for 24-well plates were coated with Matrigel (ECM550, Chemicon). LoVo and SW620 cells transfected with pGPU6/GFP/Neo-shRNA-MUC20 or pIRES2-EGFP-MUC20 were seeded in the upper chamber at 1 - 10 × 10^5^ cells per well in DMEM or RPMI 1640 serum free medium. Medium with 1% FBS was added in the bottom chamber. After 12-48 hours, the cells on the upper surface of the filter were removed using a cotton swab. The cells that had invaded through the Transwell chamber were fixed with formaldehyde and stained with 0.1% crystal violet. Ten high-power fields of each chamber were randomly selected, and the number of cells was counted using an inverted microscope.

### Western blotting analysis

Whole cell lysates were generated using RIPA lysis buffer (Abcam). Total protein samples were separated using 10% SDS-PAGE gel electrophoresis and then transferred onto a nitrocellulose membrane. The membrane was incubated with primary antibody at 4°C overnight followed by horseradish peroxidase-conjugated secondary antibody the next day for 2 hours at room temperature. The immunoreactive bands were visualized using enhanced chemiluminescence with ECL reagents (Pierce). A β-actin antibody was used as a loading control.

### Statistical analysis

The results were presented as the mean ± SEM. The data were subjected to Student’s test unless otherwise specified (χ2 test). The Kaplan-Meier survival curves and Log-rank test were used to compare survival rates. *P* values less than 0.05 were considered statistically significant.

## Results

### Identification of MUC20 from gene expression profiling

**Figure 1:**
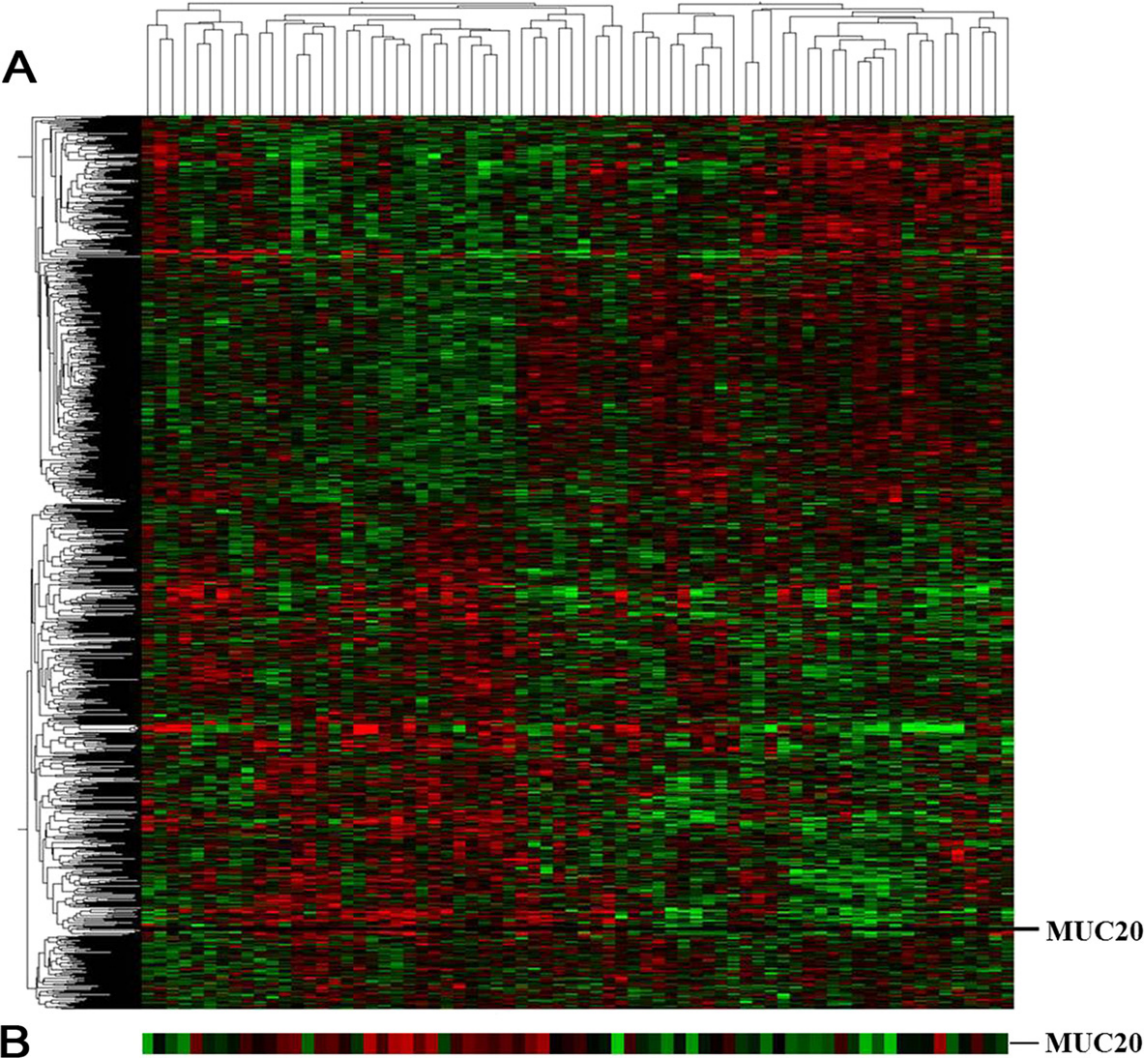
**Displays the gene expression profiling of 81 CRC tissues. (A)** A view of 887 significantly up-expressed genes and 649 significantly down-expressed genes by hierarchical clustering analysis. Each column represents a sample, and each row represents a gene. Gene expression is depicted in red (high expression) and green (low expression), respectively. Patients with poor prognosis mainly focus on the left side, and patients with good prognosis mainly focus on the right side. **(B)** Expanded view of MUC20 cluster.

Gene expression profiling of 81 CRC tissues were analyzed using a cDNA based microarray. By using random variance model (RVM), we identified 887 significantly up-expressed genes and 649 significantly down-expressed genes at significant levels (*P* < 0.05) between patients with good prognosis and poor prognosis (Table 1). A supervised hierarchical cluster analysis was performed as shown in Figure 1. Many mucins were abnormally expressed in adenocarcinomas and were associated with carcinogenesis, tumor invasion, and prognosis, as mentioned before, and we selected MUC20 (fold change =1.577, *P =* 0.005, Table 1) as a newly recognized biomarker to investigate its role in CRC.

**Table 1:** Part of the differentially up-regulated genes

**Probe set ID**	**Gene symble**	**Gene name**	***P *value**	**FDR**^**a**^	**FC**^**b**^
226654_at	MUC12	Mucin 12, cell surface associated	0.001	0.000	2.493
206700_s_at	JARID1D	Jumonji, AT rich interactive domain 1D	0.049	0.012	1.914
204351_at	S100P	S100 calcium binding protein P	0.020	0.005	1.881
231814_at	LOC100130716 /// MUC12	Similar to mucin 11 /// mucin 12, cell surface associated	0.020	0.005	1.799
203815_at	GSTT1	Glutathione S-transferase theta 1	0.045	0.011	1.780
228492_at	LOC100130216 /// USP9Y	Hypothetical protein LOC100130216 /// ubiquitin specific peptidase 9, Y-linked (fat facets-like, Drosophila)	0.028	0.007	1.770
204885_s_at	MSLN	Mesothelin	0.038	0.009	1.753
207808_s_at	PROS1	Protein S (alpha)	0.001	0.000	1.707
223646_s_at	CYorf15B	Chromosome Y open reading frame 15B	0.036	0.009	1.690
228232_s_at	VSIG2	V-set and immunoglobulin domain containing 2	0.017	0.003	1.673
205174_s_at	QPCT	Glutaminyl-peptide cyclotransferase	0.033	0.008	1.629
236518_at	KIAA1984	KIAA1984	0.001	0.000	1.614
214774_x_at	TOX3	TOX high mobility group box family member 3	0.025	0.006	1.593
203021_at	SLPI	Secretory leukocyte peptidase inhibitor	0.034	0.008	1.581
215108_x_at	TOX3	TOX high mobility group box family member 3	0.021	0.005	1.578
226622_at	MUC20	Mucin 20, cell surface associated	0.005	0.001	1.577
228821_at	ST6GAL2	ST6 beta-galactosamide alpha-2,6-sialyltranferase 2	0.010	0.002	1.565
206624_at	LOC100130216 /// USP9Y	Hypothetical protein LOC100130216 /// ubiquitin specific peptidase 9, Y-linked (fat facets-like, Drosophila)	0.031	0.008	1.565
242414_at	QPRT	Quinolinate phosphoribosyltransferase	0.028	0.007	1.560
204044_at	QPRT	Quinolinate phosphoribosyltransferase	0.045	0.011	1.560
214983_at	TTTY15	Testis-specific transcript, Y-linked 15	0.026	0.007	1.559
216623_x_at	TOX3	TOX high mobility group box family member 3	0.025	0.006	1.555

^a^: *FDR* false diagnosis rate. *P* value and FDR was calculated by the modified t test using random variance model (RVM-t test). ^b^: *FC* fold change, the ratio of probe signal in patients with poor prognosis and good prognosis.

### Up-regulation of MUC20 was a predictor of poor survival in CRC

**Figure 2:**
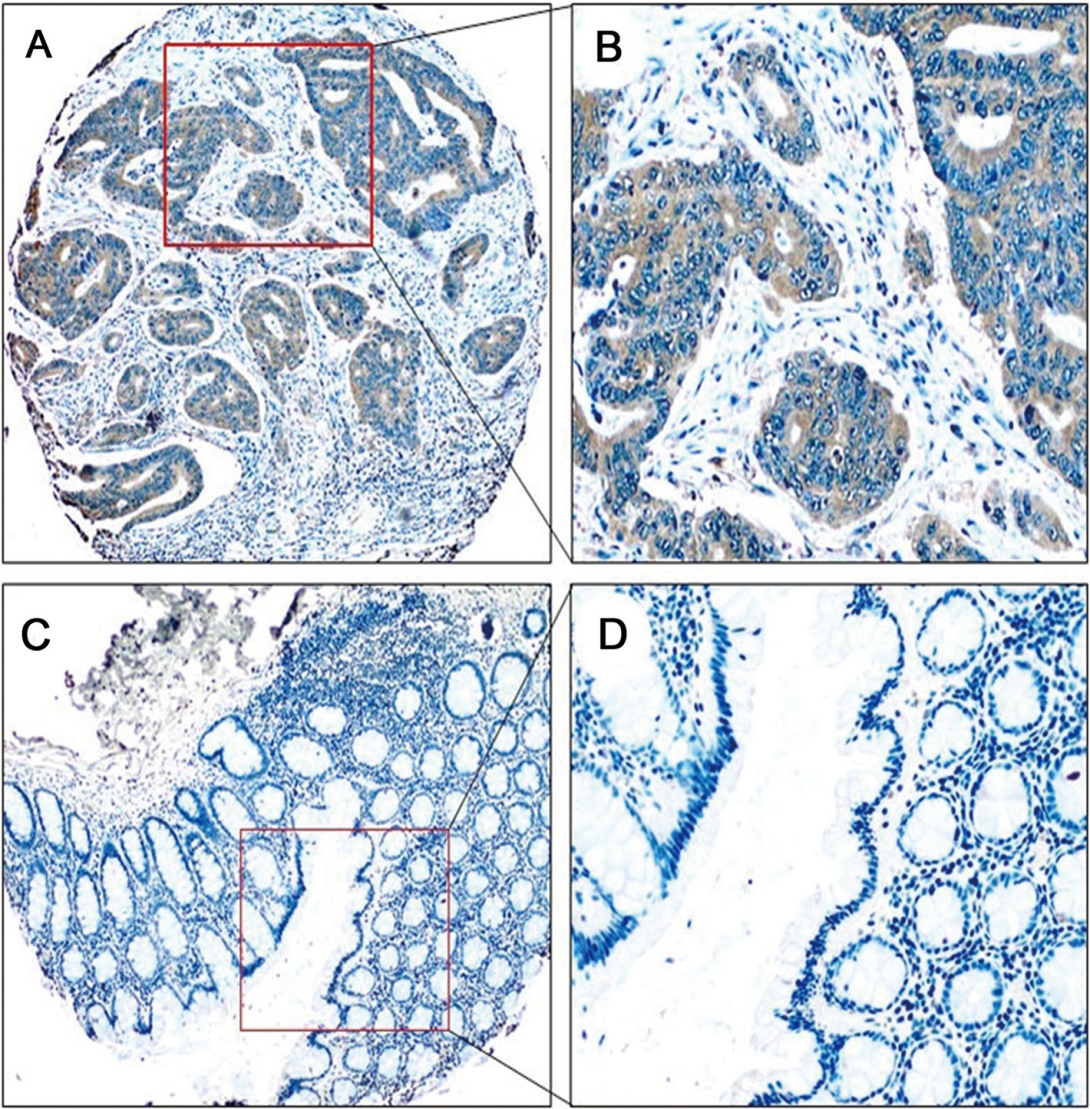
**Illustrates IHC of MUC20 in tissue microarrays (Envision × 40, ×200). (A-B)** Representative images of CRC tissues with MUC20 positive expression. **(C-D)** Representative images of ANCT with MUC20 negative expression.

Protein expression MUC20 was determined in 150 paraffin-embedded CRC tissues and ANCT using IHC. The pattern of MUC20 staining was mainly diffuse brownish cytoplasmic (Figure 2). The complete data to MUC20 expression was provided in Additional file 1: Table S1 and Table S2. The results of IHC showed that MUC20 expression in CRC tissues (60.7%, 91 of 150) was significantly higher than in ANCT (12.0%, 18 of 150, *P* < 0.05, Table 2). Table 3 summarizes the correlation of MUC20 expression with clinicopathological features. The patient ages ranged from 18 to 75 years with a median age of 55 years. In this study, 58.7% of patients were men (88 of 150) and 41.3% were women (62 of 150). Notably, up-regulation of MUC20 was correlated with recurrence (*P* = 0.016) and death (*P* = 0.015) of CRC patients. Moreover, younger patients (<60) had a higher expression of MUC20 than older patients (> = 60, *P* = 0.039). There was no relationship between MUC20 expression and gender, tumor size, location, gross type, and TNM stage.

**Table 2:** MUC20 expression in CRC tissues and ANCT

	**n**	**MUC20 expression**		**χ**^**2**^	***P***
		**Positive (%)**	**Negative (%)**		
CRC	150	91(60.7%)	59(39.3%)	76.790	0.000
ANCT	150	18(12.0%)	132(88.0%)		

**Table 3:** Correlation of MUC20 expression with clinicopathologic features in CRC patients

**Clinicopathologic features**	**n**	**MUC20 expression n (%)**		**χ**^**2**^	***P***
		**Positive**	**Negative**		
Gender				0.787	0.375
Male	88	56(63.6%)	32(26.4%)		
Female	62	35(56.5%)	27(43.5%)		
Age(years)				4.261	**0.039**
<60	94	63(67.0%)	31(33.0%)		
> = 60	56	28(50.0%)	28(50.0%)		
Tumor size (diameter)				1.189	0.276
<5	87	56(64.4%)	31(35.6%)		
> = 5	63	35(55.6%)	28(44.4%)		
Location				0.224	0.636
Colon	100	62(62.0%)	38(38.0%)		
Rectum	50	29(58.0%)	21(42.0%)		
Gross appearance				0.685	0.710
Exophytic	60	34(56.7%)	26(43.3%)		
Ulcerative	87	55(63.2%)	32(36.8%)		
Diffusely infiltrative	3	2(66.7%)	1(33.3%)		
Differentiation				0.086	0.958
High	7	4(57.1%)	3(42.9%)		
Moderate	111	67(60.4%)	44(39.6%)		
Low	32	20(62.5%)	12(37.5%)		
TNM stage				0.264	0.607
II	80	47(58.8%)	33(41.2%)		
III	70	44(62.9%)	26(37.1%)		
Recurrence				5.849	**0.016**
Yes	47	36(76.6%)	11(33.4%)		
No	90	50(55.6%)	40(44.4%)		
Status				5.960	**0.015**
Survival	93	52(55.9%)	41(44.1%)		
Death	41	32(78.0%)	9(22.0%)		

**Figure 3:**
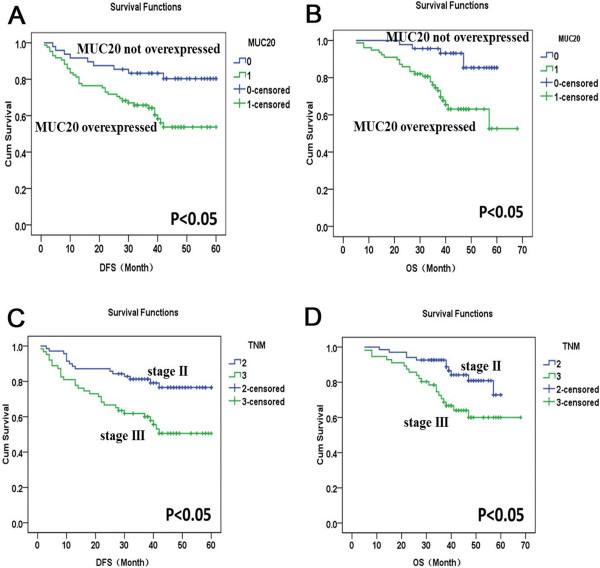
**Shows the relationship between MUC20 expression, TNM stage and DFS/OS. (A-B)** MUC20 overexpression was significantly associated with DFS and OS. 0: MUC20 not overexpressed; 1: MUC20 overexpressed. **(C-D)** TNM stage was a significant factor affecting patients’ survival. 2: stage II; 3: stage Ш.

Mean survival time of CRC patients was 42 months. The Kaplan-Meier survival curves demonstrated that the DFS and OS were significantly worse in CRC patients that overexpressed MUC20 than in patients that did not overexpress MUC20 (*P* < 0.05, Figure 3A, 3B). Univariate analysis (Log-rank test) of prognostic parameters for DFS and OS was performed. As shown in Table 4, patients that overexpressed MUC20 were significantly associated with a poorer DFS (*P* = 0.016) and OS (*P* = 0.015), stage Ш patients were also significantly associated with a poorer DFS (*P* = 0.003) and OS (*P* = 0.004) (Figure 3C, 3D). Tumor differentiation was significantly associated with a poorer OS (*P* = 0.032). Multivariate analysis using Cox’s regression model was performed as shown in Table 5. MUC20 overexpression (*P* = 0.004) and TNM stage (*P* = 0.015) were independent prognostic factors.

**Table 4:** Univariate regression model of prognostic covariates in 150 CRC patients

**Characteristics**	**DFS**		**OS**	
	**χ2**	***P***	**χ2**	***P***
Gender	0.870	0.351	0.929	0.335
Age	0.477	0.490	0.435	0.510
Location	0.122	0.727	0.216	0.642
Size	0.879	0.349	0.158	0.691
Gross type	0.173	0.917	0.486	0.784
Differentiation	1.423	0.491	4.581	**0.032**
TNM	9.061	**0.003**	8.416	**0.004**
MUC20 expression	5.849	**0.016**	5.960	**0.015**

**Table 5:** Multivariate Cox regression model for CRC patients’ survival

**Characteristics**	**Exp (B)**	**95% CI for Exp (B)**		***P***
		**Lower**	**Upper**	
Differentiation	0.439	0.054	3.547	0.440
TNM	0.400	0.191	0.839	**0.015**
MUC20 expression	0.241	0.092	0.631	**0.004**

### MUC20 mRNA expression in tissues and cells by RT-qPCR

**Figure 4:**
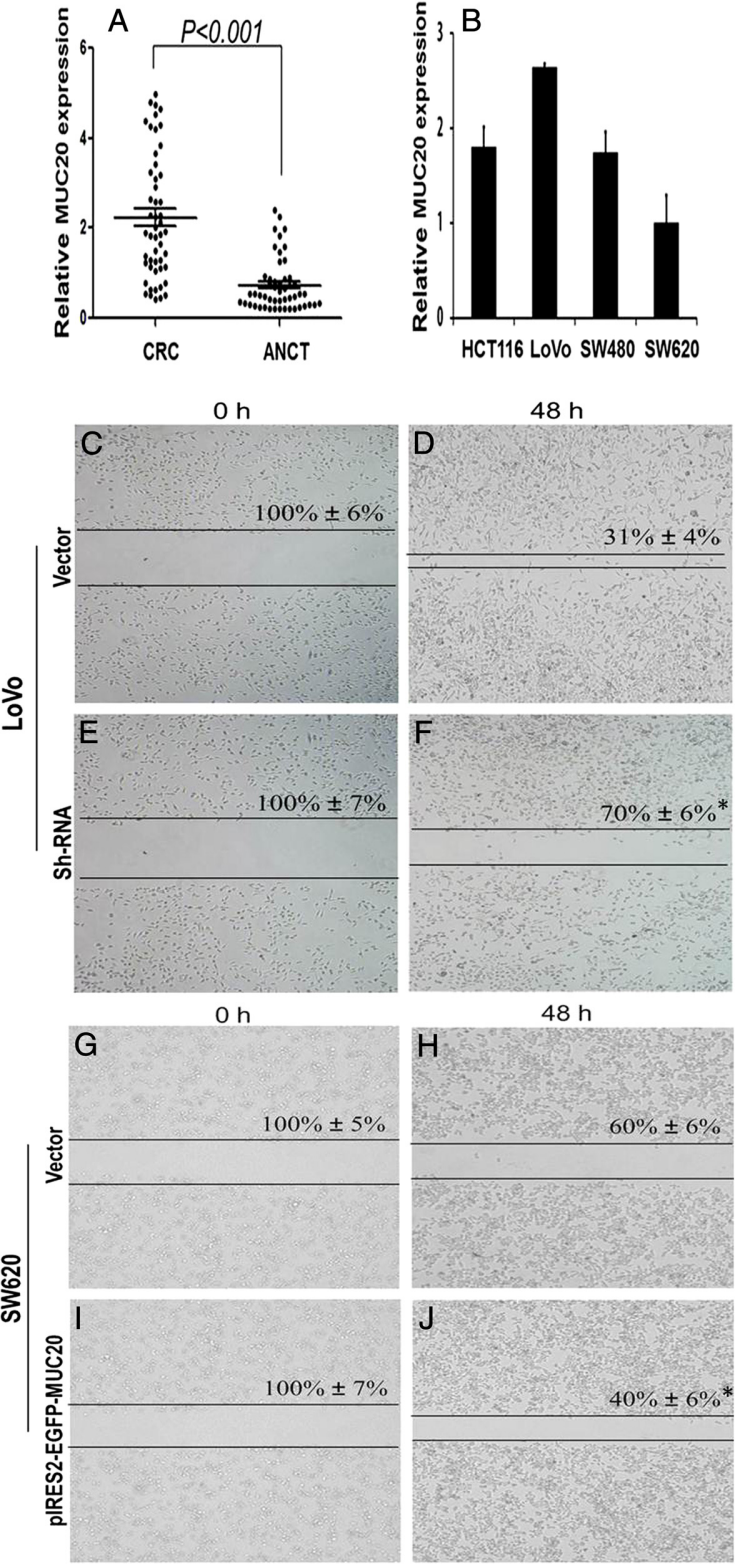
**Demonstrates MUC20 mRNA expression and cell migration in vitro (Envision × 40). (A)** Relative expression of MUC20 in CRC tissues and ANCT. The expression of MUC20 was quantified by qRT-PCR and normalized to GAPDH. **(B)** Relative expression of MUC20 in CRC cell lines. **(C-F)** Representative images of the wound healing assay with LoVo transfected with vector and shRNA. Cell migration was photographed and assessed by measuring gap sizes (inserted number represented percentage area of gap ± SD). **(G-J)** Representative images of the wound healing assay with SW620 transfected with vector and pIRES2-EGFP-MUC20. Data represent mean ± SD of triplicates. *: P < 0.05 in a comparison of the shRNA or pIRES2-EGFP-MUC20 treated group with the mock vector groups.

The mRNA amounts of MUC20 were calculated by 2^ ^(-ΔCt)^ using a relative quantification method. MUC20 mRNA expression was much higher in CRC tissues than in ANCT (*P* < 0.001, Figure 4A). The GAPDH was used as a normalization control. In addition, we analyzed the expression of MUC20 in four CRC cell lines. As shown in Figure 4B, mRNA expression of MUC20 was the highest in LoVo, the lowest in SW620, and moderate in HCT116 and SW480. Therefore, we chose LoVo for interference experiments and SW620 for overexpression experiments.

### Successful construction of Oligonucleotide and plasmid transfection

ShRNA interference sequences pGPU6/GFP/Neo-shRNA-MUC20 were designed and synthesized. The recombinant vector was confirmed to be correct by restriction enzyme digestion (BamH I and Pst I) and DNA sequencing. The overexpression plasmid pIRES2-EGFP-MUC20 was digested by the EcoRI and SalI restriction enzymes. Plasmid digestion generated two bands: a 5.3 k and a 1.5 k product were acquired in the electrophoresis lane. DNA sequencing also confirmed successful construction of the recombinant plasmid.

The transfection efficiency was monitored by green fluorescent protein (GFP) detection with converted fluorescent microscopy after 24-72 hours. The transfection efficiency was calculated as follows: GFP positive cells/total quantity of cells*100%. The transfection efficiency was the highest at 48 hours after transfection.

### MUC20 promoted migration and invasion abilities of cells in vitro

**Figure 5:**
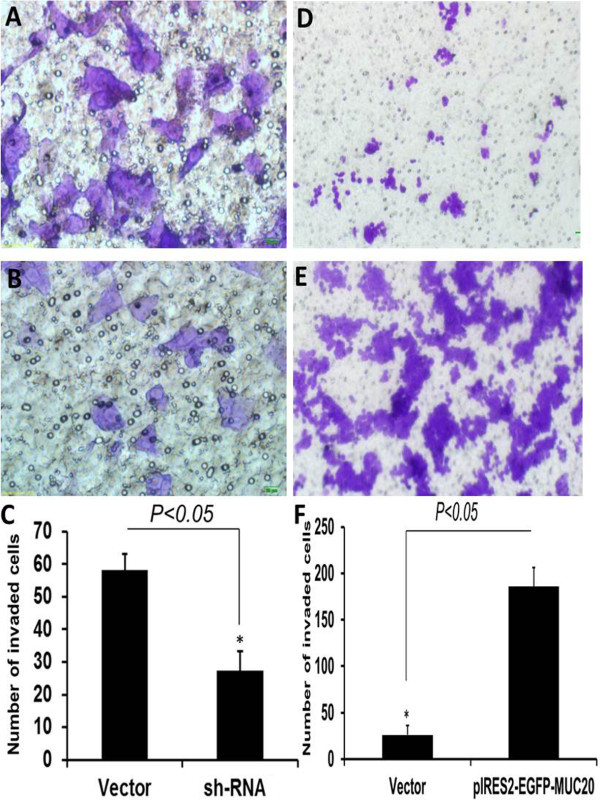
**Demonstrates influence of MUC20 expression on CRC cell invasion (Envision × 200). (A-C)** Representative images and quantification of the Transwell invasion assay with LoVo transfected with vector and shRNA. **(D-F)** Representative images and quantification of the Transwell invasion assay with SW620 transfected with vector and pIRES2-EGFP-MUC20. *:*P*<0.05.

To explore whether MUC20 affects the migration and invasion abilities of CRC cells, LoVo and SW620 cells were transfected with pGPU6/GFP/Neo-shRNA-MUC20 and pIRES2-EGFP-MUC20, respectively. The wound healing assay demonstrated that the migratory ability of LoVo cells transfected with pGPU6/GFP/Neo-shRNA-MUC20 was obviously lower than cells transfected with vector, while pIRES2-EGFP-MUC20 had the opposite effect (Figure 4C-4 J). The Matrigel Transwell invasion assay demonstrated that transfection of pGPU6/GFP/Neo-shRNA-MUC20 significantly reduced the invasive ability of LoVo cells (Figure 5, *P* < 0.05). These results suggested that MUC20 could significantly promote the migration and invasion ability of CRC cells in vitro.

### MUC20 promoted the expression of metastasis related proteins

**Figure 6:**
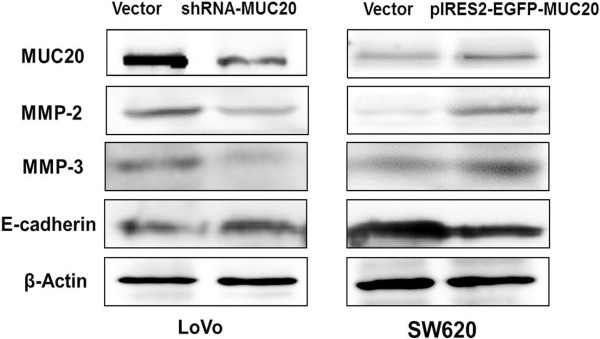
**Confirms expression levels of MUC20 and metastasis-related proteins by Western blotting.** Enforced expression of MUC20 increased MMP2 and MMP3 levels, and decreased E-cadherin level, whereas ShRNA-mediated knockdown had the opposite effect.

MMP-2, MMP-3, and E-cadherin are important metastasis related proteins in CRC [18-21]. To determine how MUC20 affects invasion and metastasis in CRC cells, we analyzed the levels of the metastasis related proteins in cells transfected with either pGPU6/GFP/Neo-shRNA-MUC20 or pIRES2-EGFP-MUC20. LoVo cells transfected with pGPU6/GFP/Neo-shRNA-MUC20 showed a significant reduction in the levels of MMP-2 and MMP-3 and a significant increase in the level of E-cadherin. SW620 cells transfected with pIRES2-EGFP-MUC20 showed the opposite effect of the transfection in LoVo cells (Figure 6). This result indicated that MUC20 could affect the expression of metastasis related proteins, which may further influence invasion and metastasis abilities of CRC cells.

## Discussion

To date, CRC studies have not directly addressed the issue of accurate prognosis for patients [22-24]. Gene expression profiling is an innovative and promising approach to investigate the underlying molecular mechanisms. Here, we profiled 81 CRC tissue samples and correlated the expression profile with patients’ survival and further investigated MUC20, a new prognostic biomarker in CRC. We demonstrated that MUC20 was overexpressed in CRC compared with ANCT. Moreover, MUC20 overexpression was significantly correlated with recurrence and death of patients, implicating that MUC20 overexpression can serve as a novel prognostic marker to identify patients with poor clinical outcome. We also showed that MUC20 knockdown/overexpression in CRC cell lines inhibited/enhanced malignant phenotypes, including migration and invasion. Collectively, our novel evidence suggested that MUC20 overexpression may involve in CRC aggressive biology.

Many gene expression profiling studies on CRC have been performed in the last decade using microarray technology, mainly focusing on carcinogenesis process, prognosis prediction, and treatment response prediction [25-27]. Studies on prognosis prediction aim to identify specific alterations to the gene expression profile that may be useful to discriminate high risk from low risk CRC [28]. Arango et al. identified a 17-gene signature that divides Dukes’C patients into two groups with significant different DFS after surgery, and found RHOA was a prognostic marker that could be used to identify a subset of patients with poor prognosis who could benefit from more aggressive treatment [29]. Yamasaki et al. performed gene expression profiling on 28 primary CRC using 119 genes differently expressed between synchronously or metachronously metastasized CRC and liver metastases, and divided tumors into two classes, localized and metastasized [30]. Our expression microarray experiments identified 887 significantly up-expressed genes and 649 significantly down-expressed genes from patients with good and poor prognosis. Most of these genes (MUC12, S100P, GSTT1, USP9Y, MSLN and so on) are involved in tumor initiation, progression or metastasis. Among these differentially expressed genes, MUC20 stood out: it has been further elucidated as a newly recognized prognostic biomarker in CRC.

Overexpression of mucins by tumor cells promotes invasion and metastases. A relationship between mucin overexpression and poor survival was found in many human tumors, including ovarian cancer [31], non-small cell lung cancer [32], and gastric cancer [33]. Previous studies showed aberrant expression of MUC1, MUC2, and MUC5AC in CRC [34-36]. Our work is the first to show that high expression of MUC20 predicts poor clinical outcome of CRC patients. Our findings were consistent with recent studies. MUC20 overexpression predicts poor prognosis in endometrial cancer and enhances EGF-triggered invasive behavior through activation of EGFR–STAT3 pathway [37]. In salivary gland carcinosarcoma, specific amplifications of MUC20 (in mesenchymal element) were observed using oligonucleotide microarray-based comparative genomic hybridization, implying its important roles in the development of carcinosarcomas [38].

Matrix metalloproteinases (MMPs) are zinc-dependent proteolytic enzymes involved in every step of tumor metastasis, including tumor growth, migration, host immune escape, extravasation, angiogenesis, and tumor invasion [39]. Specifically, high expression levels of certain MMPs, including MMP2 and MMP3, are involved in the progression, invasion and metastasis of CRC [40-42]. E-cadherin plays an essential role in the maintenance of the normal structure and cell adhesion and is associated with tumor invasion, and metastasis [43,44]. Reduced or absent E-cadherin expression has been reported in CRC [45]. To validate the effect of MUC20 on cell invasion and metastasis, we successfully constructed the recombinant plasmids for shRNA interference and overexpression experiments. Elevated expression of MUC20 promoted metastasis of CRC cells, whereas knockdown of MUC20 attenuated migration and invasion abilities of CRC cells. We further studied the effects of MUC20 interference and overexpression on metastasis related proteins. The results showed that changes in expression of MMP2 and MMP3 were consistent with MUC20, whereas E-cadherin was the opposite. We postulated that MUC20 might be involved in CRC aggressive biology.

Limitations of this study are the lack of larger-scale studies due to the small number of available biological material and the lack of comparison and integration with other similar studies. Solving these limitations should be the main goal in the future, in order to achieve the translation of promising results into clinical practice.

## Conclusion

In summary, this study provided a novel insight into the role of MUC20 in CRC. As a newly identified biomarker, MUC20 may serve as an important predictor of recurrence and poor outcome for CRC patients. MUC20 overexpression could enhance migration and invasion abilities of CRC cells. Translation of its roles into clinical practice will require further investigation.

## Abbreviations

CRC: Colorectal cancer; MUC20: Mucins 20; IgAN: IgA nephropathy; TMA: Tissue microarray; FFPE: Formalin-fixed paraffin-embedded; SI: Staining index.

## Competing interests

The authors declare that they have no competing interests.

## Authors’ contributions

XZ and XD conceived and designed the study. XX and LW performed the experiments. XX, LW, PW, and YC analyzed the data. PW, QW, SN, CT, WS, and MS contributed reagents, materials, and analysis tools. LW and XX wrote the paper. All authors read and approved the final manuscript.

## Supplementary Material

Additional file 1: Table S1 Absolute and relative frequencies of staining index (SI) in CRC tissues and ANCT. **Table S2** Correlation of MUC20 expression with clinicopathologic features in CRC patients Click here for file
