# Supplementary material for: Role of MUC20 overexpression as a predictor of recurrence and poor outcome in colorectal cancer
Source: J Transl Med. 2013 Jun 20;11:151. doi: 10.1186/1479-5876-11-151 (PMC3702436; doi:10.1186/1479-5876-11-151)
Supplement: Additional file 1: Table S1 — Absolute and relative frequencies of staining index (SI) in CRC tissues and ANCT. Table S2 Correlation of MUC20 expression with clinicopathologic features in CRC patients [file 1479-5876-11-151-S1.docx]

**Table 1 Absolute and relative frequencies of staining index (SI) in CRC tissues and ANCT**

|  | MUC20 SI | | | | | | |
| --- | --- | --- | --- | --- | --- | --- | --- |
|  | 0 | 1 | 2 | 3 | 4 | 6 | 9 |
| CRC | 48(32%) | 5(3.3%) | 6(4%) | 21(14%) | 15(10%) | 45(30%) | 10(6.7%) |
| ANCT | 118(78.7) | 4(2.7%) | 10(6.7%) | 7(4.7%) | 6(4%) | 5(3.3%) | 0 |

**Table 2 Correlation of MUC20 expression with clinicopathologic features in CRC patients**

|  |  | MUC20 intensity | | | |  | |
| --- | --- | --- | --- | --- | --- | --- | --- |
| Clinicopathologic features | n | 0 | 1+ | 2+ | 3+ | χ^2^ | *P* |
| Gender |  |  |  |  |  | 3.428 | 0.330 |
| Male | 88 | 29 | 16 | 34 | 9 |  |  |
| Female | 62 | 20 | 16 | 24 | 2 |  |  |
| Age (years) |  |  |  |  |  | 8.256 | **0.041** |
| <60 | 94 | 23 | 24 | 39 | 8 |  |  |
| >=60 | 56 | 26 | 8 | 19 | 3 |  |  |
| Tumor size (diameter) |  |  |  |  |  | 2.756 | 0.431 |
| <5 | 87 | 24 | 20 | 37 | 6 |  |  |
| >=5 | 63 | 25 | 12 | 21 | 5 |  |  |
| Location |  |  |  |  |  | 0.508 | 0.917 |
| Colon | 100 | 31 | 22 | 39 | 8 |  |  |
| Rectum | 50 | 18 | 10 | 19 | 3 |  |  |
| Gross appearance |  |  |  |  |  | 9.974 | 0.126 |
| Exophytic | 60 | 22 | 14 | 23 | 1 |  |  |
| Ulcerative | 87 | 26 | 16 | 35 | 10 |  |  |
| Diffusely infiltrative | 3 | 1 | 2 | 0 | 0 |  |  |
| Differentiation |  |  |  |  |  | 4.223 | 0.647 |
| High | 7 | 3 | 2 | 2 | 0 |  |  |
| Moderate | 111 | 34 | 26 | 44 | 7 |  |  |
| Low | 32 | 12 | 4 | 12 | 4 |  |  |
| TNM stage |  |  |  |  |  | 1.780 | 0.619 |
| II | 80 | 28 | 16 | 32 | 4 |  |  |
| III | 70 | 21 | 16 | 26 | 7 |  |  |
| Recurrence |  |  |  |  |  | 9.796 | **0.020** |
| Yes | 47 | 11 | 6 | 23 | 7 |  |  |
| No | 90 | 31 | 24 | 31 | 4 |  |  |
| Status |  |  |  |  |  | 11.030 | **0.012** |
| Survival | 93 | 32 | 24 | 34 | 3 |  |  |
| Death | 41 | 9 | 6 | 19 | 7 |  |  |
